# Effect of Prone Positioning With Individualized Positive End-Expiratory Pressure in Acute Respiratory Distress Syndrome Using Electrical Impedance Tomography

**DOI:** 10.3389/fphys.2022.906302

**Published:** 2022-06-30

**Authors:** Liangyu Mi, Yi Chi, Siyi Yuan, Huaiwu He, Yun Long, Inéz Frerichs, Zhanqi Zhao

**Affiliations:** ^1^ State Key Laboratory of Complex Severe and Rare Disease, Department of Critical Care Medicine, Peking Union Medical College Hospital, Peking Union Medical College, Chinese Academy of Medical Sciences, Beijing, China; ^2^ Department of Anesthesiology and Intensive Care Medicine, University Medical Center of Schleswig-Holstein Campus Kiel, Kiel, Germany; ^3^ Department of Biomedical Engineering, Fourth Military Medical University, Xi’an, China; ^4^ Institute of Technical Medicine, Furtwangen University, VS-Schwenningen, Germany

**Keywords:** acute respiratory distress syndrome, positive end-expiratory pressure, prone positioning, electrical impedance tomography, body mass index

## Abstract

**Background:** Positive end-expiratory pressure (PEEP) optimization during prone positioning remains under debate in acute respiratory distress syndrome (ARDS). This study aimed to investigate the effect of prone position on the optimal PEEP guided by electrical impedance tomography (EIT).

**Methods:** We conducted a retrospective analysis on nineteen ARDS patients in a single intensive care unit. All patients underwent PEEP titration guided by EIT in both supine and prone positions. EIT-derived parameters, including center of ventilation (CoV), regional ventilation delay (RVD), percentage of overdistension (OD) and collapse (CL) were calculated. Optimal PEEP was defined as the PEEP level with minimal sum of OD and CL. Patients were divided into two groups: 1) Lower Optimal PEEP_PP_ (LOP), where optimal PEEP was lower in the prone than in the supine position, and 2) Not-Lower Optimal PEEP_PP_ (NLOP), where optimal PEEP was not lower in the prone compared with the supine position.

**Results:** Eleven patients were classified as LOP (9 [8-9] vs. 12 [10-15] cmH_2_O; PEEP in prone vs. supine). In the NLOP group, optimal PEEP increased after prone positioning in four patients and remained unchanged in the other four patients. Patients in the LOP group had a significantly higher body mass index (26 [25-28] vs. 22 [17-25] kg/m^2^; *p* = 0.009) and lower ICU mortality (0/11 vs. 4/8; *p* = 0.018) compared with the NLOP group. Besides, PaO_2_/FiO_2_ increased significantly during prone positioning in the LOP group (238 [170-291] vs. 186 [141-195] mmHg; *p* = 0.042). CoV and RVD were also significantly improved during prone positioning in LOP group. No such effects were found in the NLOP group.

**Conclusion:** Broad variability in optimal PEEP between supine and prone position was observed in the studied ARDS patients. Not all patients showed decreased optimal PEEP during prone positioning. Patients with higher body mass index exhibited lower optimal PEEP in prone position, better oxygenation and ventilation homogeneity.

## Introduction

Acute respiratory distress syndrome (ARDS) presents as acute hypoxemia with bilateral pulmonary infiltrates on chest imaging, which is not fully explained by heart failure or fluid overload (Sweeney and McAuley, 2016). It occurs in approximately 10% of all ICU admissions and has a mortality of about 40% (Bellani et al., 2016). Management of ARDS is mostly supportive, and is focused on protective mechanical ventilation, prone positioning (PP) or even extracorporeal membrane oxygenation (ECMO) (Griffiths et al., 2019).

PP ventilation provides many physiological advantages for the management of patients with ARDS, including removal of the weight of the heart and mediastinum from the lung, alveolar ventilation improvement, shunt reduction with increased oxygenation, transpulmonary pressure improvement, lung strain improvement and reduction of pulmonary inflammatory cytokine production (Gattinoni et al., 2003; Kumaresan et al., 2018; Mezidi et al., 2018; Scaramuzzo et al., 2020a; Lee et al., 2020; Menk et al., 2020). The PEEP optimization during PP in ARDS remains under debate, as patients’ response varies widely among individuals. A previous study demonstrated that PP may reduce chest wall compliance, potentially necessitating higher PEEP to offset this effect. However, a recent study suggested that optimal PEEP was significantly lower in PP than that in supine position (Franchineau et al., 2020).

Electrical impedance tomography (EIT) as a non-invasive, radiation-free imaging tool that has received great interest in the respiratory management of critically ill patients (Sella et al., 2021). EIT generates cross-sectional images of the impedance distribution within the thorax and measures continuously regional lung volume changes at the bedside (Hinz et al., 2003; Kotani et al., 2016; Aguirre-Bermeo et al., 2018; Holanda et al., 2018). EIT is widely used in lung ventilation assessment in hypoxemia patients, including the PEEP titration (Dalla Corte et al., 2020; He et al., 2021; He et al., 2020; Yun et al., 2016; Becher et al., 2021; Gibot et al., 2021; Muders et al., 2021; Scaramuzzo et al., 2020b).

Since it was unclear whether PEEP in PP could be directly selected according to the PEEP in supine position, this study aimed to investigate the correlation and effect of individualized PEEP in PP compared to that in the supine position in ARDS. The primary outcome was the change of optimal PEEP between the supine and prone positions, and the secondary outcomes were the changes in lung mechanics, blood gasses and EIT-based parameters.

## Methods

A retrospective study was conducted on ARDS patients in a single ICU of Peking Union Medical College Hospital during August 2018 and November 2021. Inclusion criteria were diagnosis of ARDS according to the Berlin definition (Ranieri et al., 2012) and clinical decision to titrate optimal PEEP in both the supine and prone positions. The time interval between the two PEEP titrations was within 24 h and on average 16 h. Exclusion criteria were age <18 years and PEEP titration failed due to signal interference or spontaneous breathing. This retrospective study was approved by the Institutional Research and Ethics Committee of Peking Union Medical College Hospital. Informed consent was waived because of the retrospective nature of the study.

The patient database included demographic data, ARDS etiology, Acute Physiology and Chronic Health Evaluation (APACHE) II score, Sequential Organ Failure Assessment (SOFA) score, clinical ventilation parameters and arterial blood gas analysis. Ventilation parameters included tidal volume (V_T_), respiratory rate (RR), Respiratory system compliance (Crs), fraction of inspired oxygen (FiO_2_), PaO_2_/FiO_2_ and arterial carbon dioxide partial pressure (PaCO_2_). These data were obtained about 2 h after PEEP titration as indicated by the Intensive Care System. The baseline PEEP was set by the attending clinician according to the lower PEEP/FiO_2_ table. Outcome measurements including ICU length of stay, ICU mortality and hospital mortality were also recorded.

## EIT Data Acquisition

EIT measurements were conducted during the PEEP titration periods in both supine and prone positions with PulmoVista 500 (Dräger Medical, Lübeck, Germany). An EIT belt with 16 electrodes was placed around the patient’s thorax at the 4-fifth intercostal space level.

The following EIT-based parameters were calculated in the study: center of ventilation (CoV), global inhomogeneity index (GI), regional ventilation delay (RVD), overdistension (OD), collapse (CL) and ventilation distribution at each PEEP level. Lung images were divided into two symmetrical non-overlapping ventral and dorsal horizontal regions of interest (ROIs).

The CoV describes the weighted geometrical center of the ventilation distribution (Frerichs et al., 1998). The CoV value increases when regional tidal ventilation is distributed preferentially towards the gravity-dependent lung region.

The GI index was used to quantify the tidal volume distribution within the lung (Zhao et al., 2010). A lower GI index value indicated a more homogeneous ventilation.

RVD is defined as the time delay of regional impedance time curve to reach a certain threshold (Wrigge et al., 2008). The RVD correlates well with tidal recruitment/derecruitment.

Costa et al. proposed an EIT-based algorithm that estimates cumulated alveolar collapse and overdistension during PEEP titration (Costa et al., 2009). The high initial PEEP levels lead to lung hyperdistension (OD), which can be assessed as a percent decrease in pixel compliance in relation to its peak value (best pixel compliance) measured at lower PEEPs. Similarly, recruitable alveolar collapse (CL) can be estimated at lower PEEPs against the best pixel compliance. Previous randomized controlled trials (total n > 200) suggested that PEEP titration using OD and CL information resulted in better clinical outcomes (He et al., 2021; Hsu et al., 2021). The limitations of the method were discussed in a previous study (Zhao et al., 2020) and considered in the clinical practice.

## Optimal PEEP by EIT

Firstly, we perform 2 min of lung recruitment according to the patient’s condition, the following three different levels of lung recruitment pressure can be selected: A. PC 15 cmH_2_O+ PEEP 24 cmH_2_O (for patients with P/F < 100 mmHg); B. PC 15 cmH_2_O+ PEEP 21 cmH_2_O (for patients with 100 ≤ P/F < 200 mmHg); C. PC 15 cmH_2_O+ PEEP 18 cmH_2_O (for patients with 200 ≤ P/F < 300 mmHg); FiO_2_ adjusted to 100% during recruitment. If the initial clinical judgment cannot tolerate the pressure RM of A or B, the pressure RM of the lower pressure B or C can be selected. PEEP was increased to 21 cmH_2_O, if the baseline PEEP was higher than 10 cmH_2_O and the patient tolerated the increase, as assessed by the physician (e.g., absence of impaired circulation). Otherwise, PEEP of 15 cmH_2_O was used. A decremental PEEP trial was performed starting from 21 or 15 cmH_2_O and decreasing to 0 cmH_2_O in 2-min steps of three cmH_2_O in supine position. OD and CL were estimated based on the decrease of regional respiratory compliance curve during the decremental PEEP trial. EIT images were recorded in every PEEP level. Optimal PEEP values were determined based on the minimum sum of OD and CL. Repeat lung recruitment after titration of PEEP. The optimal PEEP maintained for about 10 h in supine position. Subsequently, patients were turned to prone position. PEEP titration was conducted using the same procedure 1–2 h after prone position, and the optimal PEEP was maintained for about 14 h during prone position.

According to the levels of optimal PEEP in supine and prone positions, patients were divided into two groups: 1) Lower Optimal PEEP_PP_ (LOP), where optimal PEEP was lower in the prone than in the supine position, and 2) Not-Lower Optimal PEEP_PP_ (NLOP), where optimal PEEP was equal or higher in the prone position compared with in the supine position.

## Statistical Analysis

Statistical analyses were computed with Prism 8.0.2 software (GraphPad Software, San Diego, CA) and the SPSS 24.0 software package (SPSS Inc., Chicago, IL, United States). The results were expressed as mean ± standard deviation or median (25th-75th percentile) for continuous variables, and numbers (percentages) for categorical variables. Differences between positions and groups were compared by using the *t* test or the Wilcoxon signed rank test where appropriate. Chi-square and Fisher’s exact tests were used to compare categorical variables. ANOVA for repeated measures was used to compare data obtained at multiple PEEP levels, followed by pairwise comparisons using a Dunn post hoc test with Bonferroni correction. *p* < 0.05 was considered statistically different.

## Results

### Population Characteristics and Outcome Between the Two Groups

**FIGURE 1:**
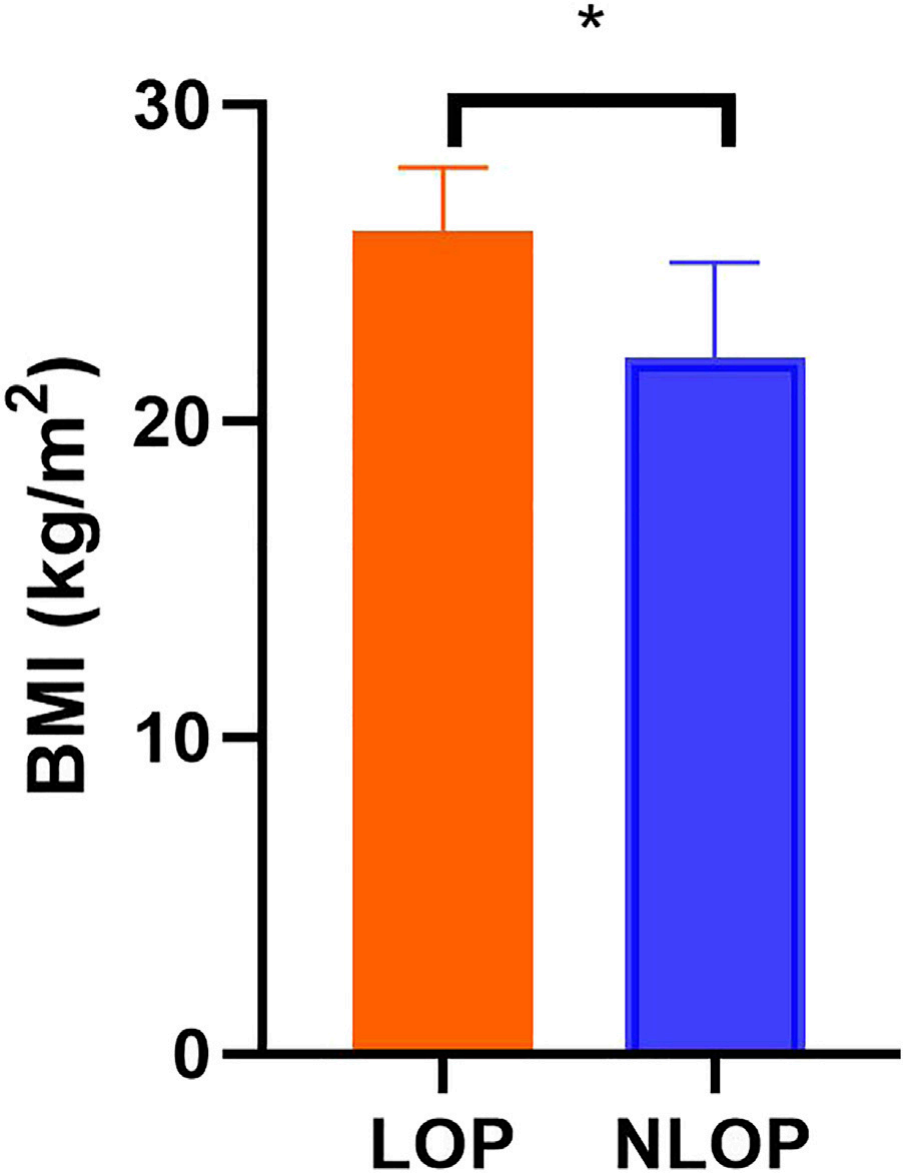
Body mass index of the two groups. Patients in the LOP group had higher body mass index compared with the NLOP group. *p < 0.05 compared with the LOP group.

Nineteen patients (age 64 [52–70] years, 37% male) were included, 11 (55%) of them in the LOP group. Their main characteristics are reported in Table 1. The cause of ARDS was most frequently of pulmonary origin (74%), and five patients were treated with ECMO. The baseline scores of APACHE II and SOFA were 19 (16, 20) and 13 (11, 14) respectively. Patients in the LOP group had higher body mass index (26 [25-28] vs. 22 [17-25] kg/m^2^; *p* = 0.009) compared with the NLOP group (Figure 1). There was no difference between the two groups in other basic population characteristics. In the LOP group, no patient died during the ICU stay. However, 4 (50%) patients died in the NLOP group (*p* = 0.018). Hospital mortality was also lower in the LOP group (9% vs. 75%; *p* = 0.006) (Table 1).

**TABLE 1:** Characteristics and outcomes.

Characteristic	Total (*n* = 19)	NLOP (*n* = 8)	LOP (*n* = 11)	*p* Value
Age, year	64 (52, 70)	70 (53, 73)	63 (52, 68)	0.342
Male, n (%)	7 (37)	3 (38)	4 (36)	1.000
BMI (kg/m^2^)	25 (21, 27)	22 (17, 25)	26 (25, 28)	0.009
BMI (kg/m2)-no ECMO	24 (19, 26)	21 (17.25)	26 (23, 29)	0.018
APACHE II	19 (16, 20)	18 (18, 19)	20 (14, 24)	0.648
SOFA	13 (11, 14)	14 (11, 14)	12 (10, 14)	0.560
ARDS-risk factor				0.338
Extrapulmonary	5 (26)	1 (12)	4 (36)	
Pulmonary	14 (74)	7 (88)	7 (64)	
Lesion				1.000
Diffuse, n (%)	8 (42)	3 (38)	5 (45)	
Focal, n (%)	11 (58)	5 (62)	6 (55)	
Grade				0.367
Mild	6 (32)	4 (50)	2 (18)	
Moderate	12 (63)	3 (38)	9 (82)	
Severe	1 (5)	1 (12)	0 (0)	
ECMO, n (%)	5 (26)	1 (12)	4 (36)	0.338
ICU length of stay (d)	32 (16, 44)	32 (18, 77)	32 (16, 44)	0.756
In-ICU mortality (%)	4 (21)	4 (50)	0 (0)	0.018
Hospital mortality (%)	7 (37)	6 (75)	1 (9)	0.006

LOP, the patients whose optimal PEEP was lower in the prone than in the supine position; NLOP, the patients with the optimal PEEP, not lower in the prone position; BMI, body mass index; APACHE II, Acute Physiology and Chronic Health Evaluation II; SOFA, Sequential Organ-Failure Assessment; ARDS, acute respiratory distress syndrome; PEEP, positive end-expiratory pressure; ECMO, extracorporeal membrane oxygenation; ICU, intensive care unit.

### Ventilator and Respiratory Parameters in Supine Position and Prone Positions

**FIGURE 2:**
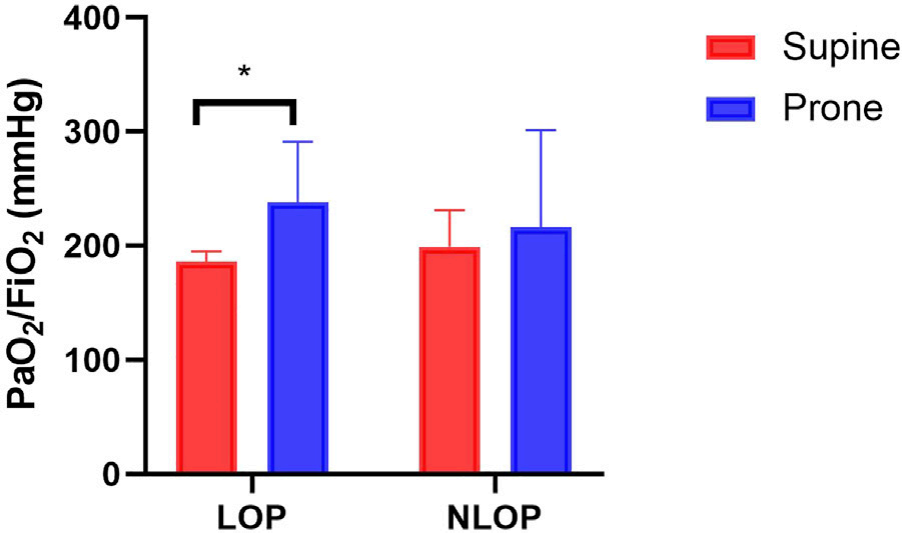
Ratio between the arterial partial pressure of oxygen and fraction of inspired oxygen (PaO_2_/FiO_2_) in different positions of the two groups. PaO_2_/FiO_2_ in the LOP group was significantly increased during prone position compared with supine position. No significant difference was observed in the NLOP group. *p < 0.05 compared with supine position.

PaO_2_/FiO_2_ in the LOP group was significantly increased during PP (238 [170-291] vs. 186 [141-195] mmHg; *p* = 0.042) (Table 2 Figure 2). No significant difference was observed between the two positions regarding V_T_, RR, Cdyn, FiO_2_ and PaCO_2_ in both groups. The ventilator parameters at baseline did not differ between the two groups as well (Table 3).

**TABLE 2:** Difference of ventilator parameters at supine position and prone position.

	Group	SP	PP	*p* Value
V_T_ (ml)	Total	382 (235, 446)	349 (224, 456)	0.570
	LOP	390 (276, 446)	370 (213, 550)	0.476
	NLOP	324 (208, 459)	332 (272, 404)	0.933
V_T_ (ml/kg)	Total	4.8 (3.5, 6.7)	5.5 (3.1, 7.6)	0.639
	LOP	4.7 (3.5, 5.9)	5.5 (3.0, 7.6)	0.811
	NLOP	6.0 (3.1, 8.5)	6.0 (3.9, 8.2)	0.985
V_T_ (ml/kg)-no ECMO	Total	6.0 (4.4, 7.4)	6.6 (5.2, 7.9)	0.358
	LOP	5.7 (4.7, 6.7)	6.4 (5.5, 7.6)	0.383
	NLOP	6.1 (2.8, 8.9)	6.7 (4.9, 8.5)	0.934
RR (bpm)	Total	17 (16, 20)	19 (16, 23)	0.440
	LOP	16 (14, 21)	16 (14, 20)	0.462
	NLOP	18 (17, 22)	22 (18, 29)	0.104
Crs (ml/cmH_2_O)	Total	16.3 (14.0, 32.4)	18.4 (16.7, 35.7)	0.066
	LOP	20.0 (14.3, 32.4)	24.7 (17.2, 39.2)	0.147
	NLOP	15.6 (12.7, 25.4)	17.9 (14.5, 33.0)	0.313
Crs (ml/cmH_2_O)-no ECMO	Total	19.4 (13.9.41.5)	28.8 (17.0.42.0)	0.456
	LOP	29.4 (16.3.40.1)	38.24 (17.3.47.7)	0.535
	NLOP	15.8 (7.5, 45.6)	18.4 (16.3.33.1)	0.205
FiO_2_ (%)	Total	45 (40, 50)	40 (38, 50)	0.035
	LOP	45 (40, 50)	40 (38, 48)	0.181
	NLOP	48 (40, 52)	48 (39, 50)	0.181
PaO_2_/FiO_2_ (mmHg)	Total	186 (141, 218)	238 (160, 298)	0.023
	LOP	186 (141, 195)	238 (170, 291)	0.042
	NLOP	199 (145, 231)	216 (151, 301)	0.313
PaO_2_/FiO_2_ (mmHg)-no ECMO	Total	185 (132,217)	250 (143,359)	0.165
	LOP	185 (132,195)	250 (149,365)	0.204
	NLOP	186 (132,224)	162 (126,352)	0.620
PaCO_2_ (mmHg)	Total	43 (39, 48)	43 (41, 50)	0.732
	LOP	42 (39, 47)	42 (41, 51)	0.286
	NLOP	47 (39, 54)	45 (43, 49)	0.575
PaCO_2_ (mmHg)-no ECMO	Total	46 (41.53)	46 (42.57)	0.692
	LOP	43 (42.47)	45 (42.59)	0.477
	NLOP	48 (40.58)	47 (41.53)	0.710
Pplat (cmH_2_O)	Total	24 (21.28)	24 (21.26)	0.491
	LOP	23 (21.28)	23 (21.26)	0.529
	NLOP	25 (18.33)	25 (19.28)	0.779
Baseline PEEP (cmH_2_O)	Total	8 (5, 10)	8 (6, 11)	0.959
	LOP	10 (6, 11)	10 (6, 11)	0.787
Optimal PEEP (cmH_2_O)	NLOP	8 (5, 8)	7 (6, 9)	0.787
	Total	9 (6, 12)	9 (6, 9)	0.116
	LOP	12 (10, 15)	9 (8, 9)	0.002
	NLOP	5 (3, 8)	6 (6, 9)	0.089
Optimal PEEP-no ECMO (cmH_2_O)	Total	9 (3, 13)	9 (6, 10)	0.886
	LOP	12 (12, 15)	9 (9.12)	0.084
	NLOP	3 (3, 6)	6 (6, 9)	0.139

LOP, the patients whose optimal PEEP, was lower in the prone than in the supine position; NLOP, the patients with the optimal PEEP, not lower in the prone position; SP, supine position; PP, prone position; EIT, electrical impedance tomography; V_T_, tidal volume; RR, respiratory rate; bpm, breaths per minute; Crs, respiratory system compliance; FiO_2_, fraction of inspired oxygen; PaO_2=_arterial partial pressure of oxygen; PaCO_2_, arterial partial pressure of carbon dioxide.

**TABLE 3:** Difference of baseline ventilator parameters in LOP and NLOP.

	LOP	NLOP	*p* Value
V_T_ (ml)	390 (276, 446)	324 (208, 459)	0.700
V_T_ (ml/kg)	4.7 (3.5, 5.9)	6.0 (3.1, 8.5)	0.395
RR (bpm)	16 (14, 21)	18 (17, 22)	0.261
Crs (ml/cmH_2_O)	20.0 (14.3, 32.4)	15.6 (12.7, 25.4)	0.657
PaO_2_/FiO_2_ (mmHg)	186 (141, 195)	199 (145, 231)	0.717
PaCO_2_ (mmHg)	43 (42.47)	48 (40.58)	0.351
Pplat (cmH_2_O)	23 (21.28)	25 (18.33)	0.951
Baseline PEEP (cmH_2_O)	10 (6, 11)	8 (5, 8)	0.498
Optimal PEEP (cmH_2_O)	12 (10, 15)	5 (3, 8)	0.001

LOP, the patients whose optimal PEEP, was lower in the prone than in the supine position; NLOP, the patients with the optimal PEEP, not lower in the prone position; SP, supine position; PP, prone position; EIT, electrical impedance tomography; VT, tidal volume; RR, respiratory rate; bpm, breaths per minute; Crs, respiratory system compliance; FiO_2_, fraction of inspired oxygen; PaO_2_, arterial partial pressure of oxygen; PaCO_2_, arterial partial pressure of carbon dioxide.

### Effect of Prone Position on EIT-Related Parameters at Different PEEP Levels

**FIGURE 3:**
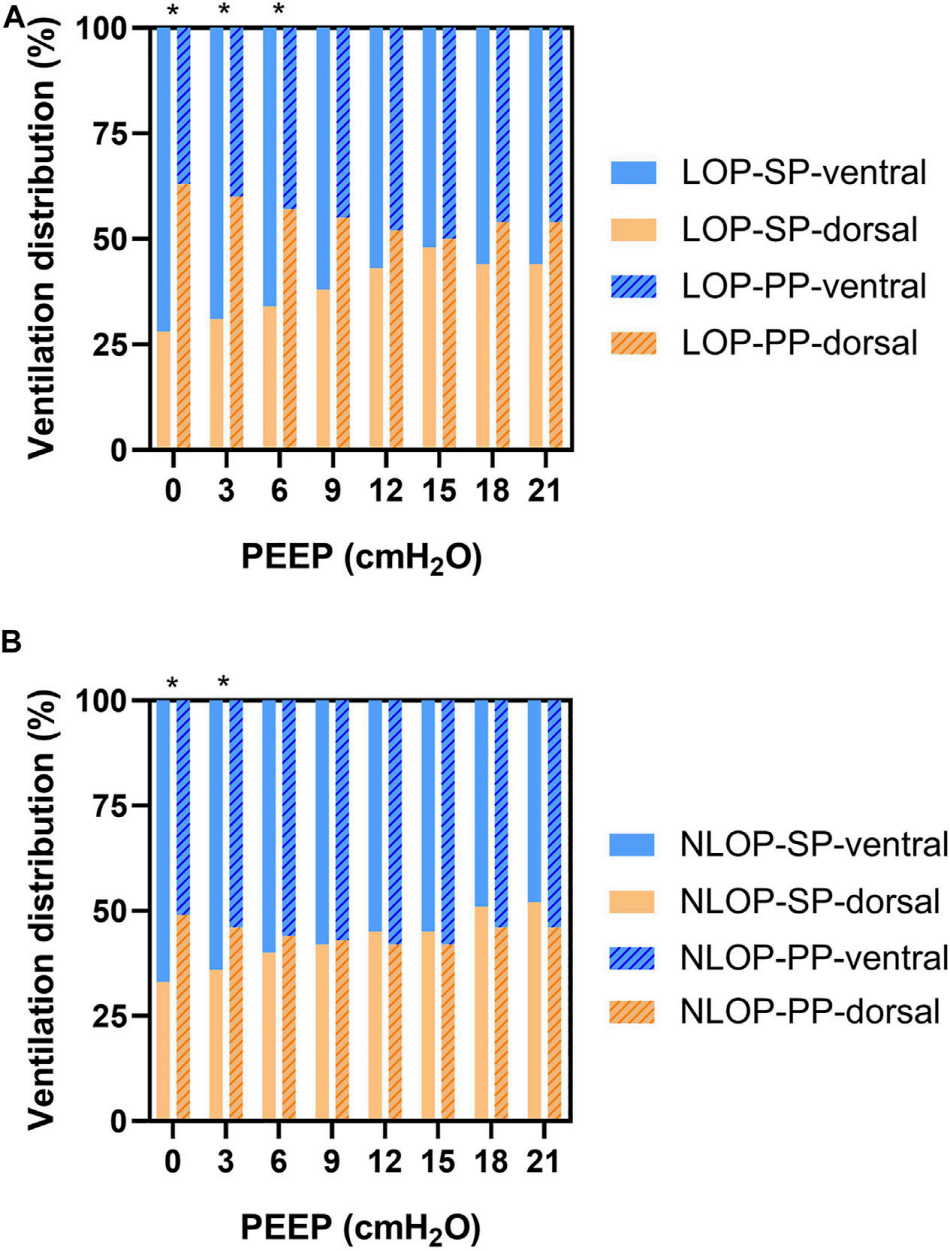
Dorsal ventilation was significantly higher during prone position at lower PEEP in the LOP group (PEEP = 0, 3, 6 cmH_2_O) **(A)**. The significant improvement of dorsal ventilation was also present at PEEP of 0 and 3 cmH_2_O in the NLOP group **(B)**. *p < 0.05 compared with supine position.

In the LOP group, dorsal ventilation was significantly increased during prone positioning at lower PEEP levels (PEEP = 0, 3, 6 cmH_2_O). The significant improvement of dorsal ventilation was also seen at 0 and 3 cmH_2_O of PEEP in the NLOP group (Figure 3). CoV was improved in the LOP group during PP at zero PEEP (41.7 ± 7.3% vs. 54.0 ± 6.3%; *p* = 0.01), PEEP = 3 cmH_2_O (42.6 ± 6.5% vs. 53.6 ± 5.2%; *p* = 0.01) and PEEP = 6 cmH_2_O (44.4 ± 7.4% vs. 52.9 ± 5.3%; *p* = 0.03); RVD was lower in the LOP group during PP at zero PEEP (6.54 ± 5.22 vs. 2.98 ± 1.36; *p* = 0.01) and PEEP = 3 cmH_2_O (4.01 ± 2.09 vs. 2.67 ± 1.10; *p* = 0.01). Similar effects were not found in the NLOP group (Table 4).

**TABLE 4:** Effect of prone position on EIT-related parameters at different PEEP levels.

			Baseline	PEEP = 21	PEEP = 18	PEEP = 15	PEEP = 12	PEEP = 9	PEEP = 6	PEEP = 3	PEEP = 0
GI	LOP	SP	0.40 ± 0.11	0.40 ± 0.03	0.37 ± 0.02	0.42 ± 0.09	0.40 ± 0.08	0.44 ± 0.08	0.50 ± 0.11	0.56 ± 0.16	0.64 ± 0.22
		PP	0.42 ± 0.24	0.40 ± 0.06	0.37 ± 0.02	0.43 ± 0.18	0.42 ± 0.18	0.43 ± 0.23	0.51 ± 0.28	0.50 ± 0.27	0.60 ± 0.35
	NLOP	SP	0.44 ± 0.11	0.41 ± 0.05	0.40 ± 0.05	0.43 ± 0.07	0.43 ± 0.07	0.44 ± 0.09	0.45 ± 0.11	0.47 ± 0.12	0.50 ± 0.13
		PP	0.42 ± 0.18	0.38 ± 0.01	0.44 ± 0.07	0.47 ± 0.16	0.46 ± 0.15	0.45 ± 0.17	0.48 ± 0.18	0.72 ± 0.63	0.57 ± 0.22
CoV	LOP	SP	46.0 ± 6.6	44.9 ± 8.2	44.4 ± 6.8	47.5 ± 7.1	47.1 ± 7.0	45.9 ± 7.1	44.4 ± 7.4	42.6 ± 6.5	41.7 ± 7.3
		PP	51.5 ± 6.4	51.3 ± 6.3	51.2 ± 5.4	50.2 ± 6.5	51.0 ± 5.9	52.2 ± 5.4	52.9 ± 5.3^*^	53.6 ± 5.2^*^	54.0 ± 6.3^*^
	NLOP	SP	48.0 ± 5.5	48.5 ± 2.8	48.1 ± 2.8	47.3 ± 4.4	47.8 ± 5.9	46.9 ± 5.7	46.1 ± 5.8	45.0 ± 6.2	44.1 ± 6.4
		PP	54.5 ± 8.6	49.0 ± 4.2	48.9 ± 4.0	50.4 ± 10.7	48.2 ± 12.1	48.7 ± 11.7	49.2 ± 11.4	49.9 ± 10.9	50.7 ± 10.5
RVD	LOP	SP	3.88 ± 1.70	1.74 ± 0.64	2.49 ± 1.52	3.02 ± 1.60	4.02 ± 2.87	4.01 ± 2.26	4.02 ± 2.37	4.01 ± 2.09	6.54 ± 5.22
		PP	3.33 ± 1.85	1.59 ± 0.60	2.54 ± 0.82	3.04 ± 1.45	2.88 ± 1.32	2.86 ± 1.40	2.81 ± 1.19	2.67 ± 1.10^*^	2.98 ± 1.36^*^
	NLOP	SP	4.55 ± 3.05	1.84 ± 1.01	1.81 ± 2.16	2.74 ± 1.25	3.24 ± 1.49	3.80 ± 1.63	3.90 ± 3.06	3.19 ± 0.93	3.78 ± 1.10
		PP	4.00 ± 2.19	1.70 ± 0.82	1.60 ± 1.03	3.59 ± 3.31	3.96 ± 3.12	4.02 ± 3.69	4.32 ± 3.40	3.92 ± 2.37	4.26 ± 2.07

PEEP, positive end-expiratory pressure; GI, global inhomogeneity index; CoV, center of ventilation; RVD, regional ventilation delay; *p < 0.05 compared with supine position.

### EIT-Titrated Optimal PEEP Between Supine and Prone Positions

**FIGURE 4:**
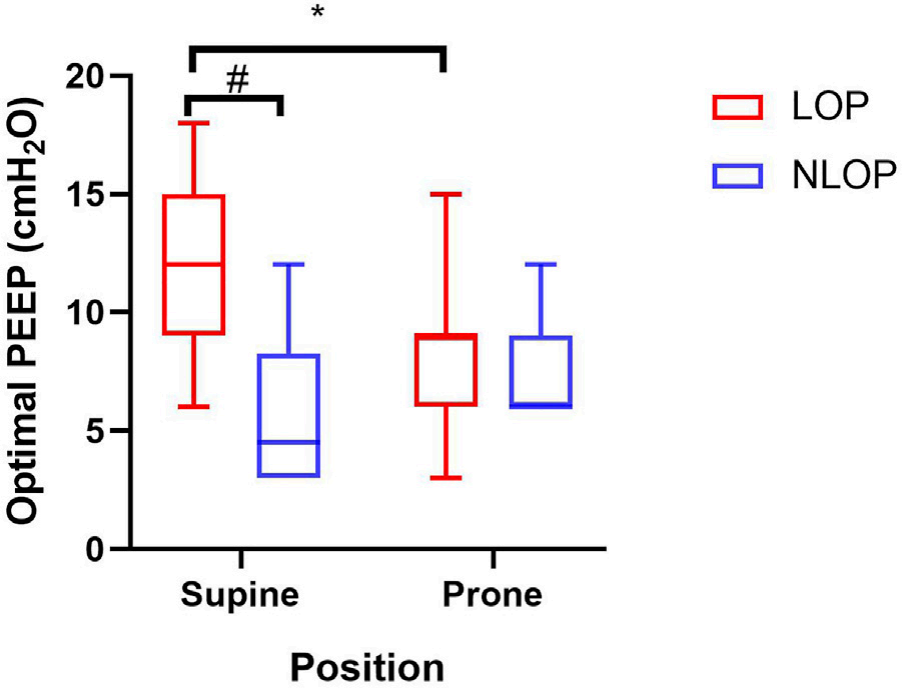
Electrical impedance tomography (EIT)-estimated optimal positive end-expiratory pressure (PEEP) in supine and prone positions. #*p* < 0.05 compared with the LOP group. *p < 0.05 compared with supine position.

In 7 cases PEEP was titrated from 21 cmH_2_O and in twelve cases from 15 cmH_2_O. Broad variability in optimal PEEP between supine and prone positions was observed in the studied patients with ARDS. There were eleven patients whose EIT-based optimal PEEP was reduced in prone position in the LOP group, the optimal PEEP shifting from 12 (10, 15) to 9 (8, 9) cmH_2_O; However, in the NLOP group, PEEP was elevated during PP in four patients and unchanged in the other four patients (Figure 4).

## Discussion

The main findings of our study can be summarized as follows: 1) Broad variability in optimal PEEP between supine and prone position was observed in the ARDS patients. Not all patients showed decreased optimal PEEP during PP. 2) Patients with lower optimal PEEP in prone position had higher body mass index and led to better oxygenation and ventilation homogeneity.

PP is currently widely applied in moderate-to-severe ARDS patients. Lung density redistributes from dorsal to ventral regions due to recruitment in dorsal lung regions and collapse of ventral ones when patients are shifted into the prone position. And some studies have shown that prone positioning can also improve transpulmonary pressure and lung stress. But the overall effect of PP is the decrease in chest wall compliance, which results in an increase in plateau pressure during volume-controlled ventilation or a decrease in V_T_ during pressure-controlled ventilation (Guerin et al., 2004; Taccone et al., 2009; Gattinoni et al., 2013; Iftikhar et al., 2015; Guérin et al., 2020; Lai et al., 2021). Recent studies suggested that EIT-based optimal PEEP was significantly lower in prone than in supine position (Kotani et al., 2018; Martinsson et al., 2021). On the contrary, a study showed that in most patients a PEEP value above commonly used settings was necessary to avoid alveolar collapse in the prone position (Spaeth et al., 2016). Therefore, the choice of PEEP in the PP is still controversial. In our study we analyzed nineteen ARDS patients whose optimal PEEP values were titrated by EIT in both supine and prone positions, and we were able to show that not all of the patients had lower optimal PEEP in prone position. The optimal PEEP was decreased in 11 patients during PP, while it increased in four patients and remained unchanged in the other four patients. Due to the broad variability in optimal PEEP between supine and prone position observed in these patients, our research suggests that an individual PEEP for prone position might not be derived from the optimal PEEP for supine position in ARDS.

A recent study suggested that optimal PEEP was significantly lower in prone than in supine position (Franchineau et al., 2020). This does not correspond with our results. Thus, the patients were separated into two groups, one of which was named the LOP group where optimal PEEP was lower in the prone than in the supine group, the remaining patients were allocated into the NLOP group. We found that the BMI of the patients in the LOP group was significantly higher than that in the NLOP group. The median BMI of patients was 29 kg/m^2^ in the study from Franchineau et al. (Franchineau et al., 2020), which was also higher than the normal range. As previous study showed, fat accumulation in chest wall and abdomen of obese patients in supine position restricted the diaphragm movement and decreased lung compliance. These factors decrease lung compliance, functional residual capacity, and increase work of breathing and airway resistance in obese patients. Furthermore, PP could be more important in obese patients because of their decreased functional residual capacity and increased atelectasis. PP can partly offset these adverse effects, and obese patients have better responsiveness and prognosis to prone position as reported in recent studies (Chergui et al., 2007; De Jong et al., 2013).

Interestingly, PaO_2_/FiO_2_ in the LOP group was significantly increased during PP. EIT-derived parameters in the LOP group, including CoV and RVD, were also improved after PP at lower PEEP levels. Similar effects were not found in patients without decrease of optimal PEEP during prone positioning. Besides, the outcomes of the two groups were quite different, the ICU and hospital mortality in the NLOP group was significantly higher than that in the LOP group. This may be related to the poor responsiveness of the latter patients to prone position. There is evidence that PaO_2_/FiO_2_ after the PP differed significantly between ICU survivors and non-survivors (Lee et al., 2020). Although we observed improvement in oxygenation and better prognosis after PP in overweight patients, the small sample size of this retrospective study does not allow to draw the conclusion that the prone position is not required in lean patients.

Our study has several limitations. First, this is a retrospective study, but the PEEP titration method in the supine and prone positions were consistent. Optimal PEEP results were thus comparable. Although the time interval between the two examinations was different, the change in posture occurred within 1 day, approximately after 16 h on average. Second, the sample size was relatively small, and only 19 patients were included. Third, there were some potential confounding factors, e, g, five patients were treated with ECMO. However, no effect on the EIT-derived parameters is presumed because of identical evaluation of EIT data. Therefore, only some preliminary conclusions have been drawn so far, and prospective studies with larger sample sizes can be conducted in the future to explore and verify the current results.

## Conclusion

Broad variability in optimal PEEP between supine and prone positions was observed in ARDS patients. Not all patients showed decreased optimal PEEP during prone positioning. Patients with lower optimal PEEP in prone position had higher body mass index and led to better oxygenation and ventilation homogeneity.

## Key Messages

1) Broad variability in optimal PEEP between supine and prone position was observed in ARDS patients. Not all patients exhibited decreased optimal PEEP during prone positioning.
2) Patients with lower optimal PEEP in prone position had higher body mass index and led to better oxygenation and ventilation homogeneity.

## Data Availability

The original contributions presented in the study are included in the article, further inquiries can be directed to the corresponding authors.

## Abbreviations

APACHE, Acute Physiology and Chronic Health Evaluation; ARDS, acute respiratory distress syndrome; BMI, body mass index; CoV, center of ventilation; Cdyn, dynamic respiratory system compliance; ECMO, extracorporeal membrane oxygenation; EIT, electrical impedance tomography; FiO_2_, fraction of inspiratory oxygen; GI, global inhomogeneity; ICU, intensive care unit; PaO_2_, arterial partial pressure of oxygen; PaCO_2_, arterial partial pressure of carbon dioxide; PEEP, positive end-expiratory pressure; PP, prone positioning; ROI, region of interest; RR, respiratory rate; RVD, regional ventilation delay; SOFA, Sequential Organ-Failure Assessment; SP, supine position; V_T_, tidal volume.
